# Supplementary material for: Pregnancy reduces COVID-19 vaccine immunity against novel variants
Source: NPJ Vaccines. 2025 Aug 13;10:191. doi: 10.1038/s41541-025-01236-4 (PMC12350772; doi:10.1038/s41541-025-01236-4)
Supplement: Supplementary file 1 — Supplementary Information [file 41541_2025_1236_MOESM1_ESM.pdf]

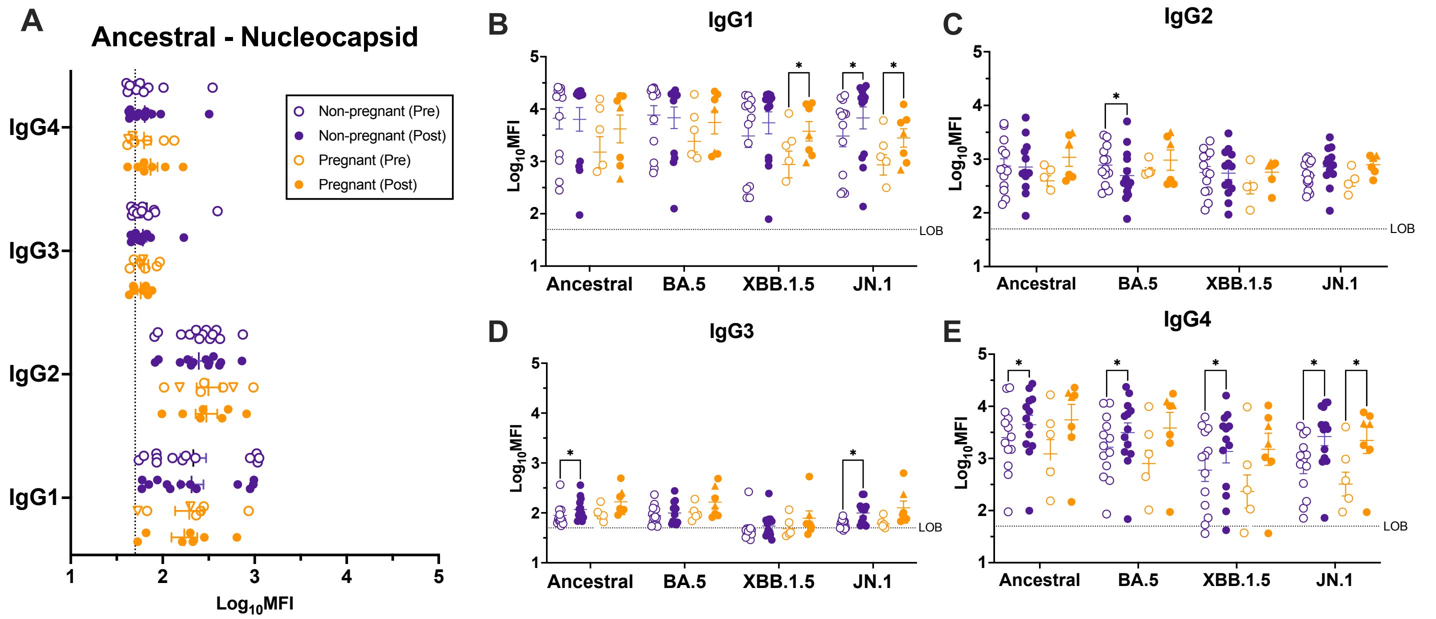

Supplemental Figure 1. Vaccination differently induces IgG subtype responses during pregnancy. IgG antibody responses in serum pre and 3-5 weeks post-vaccination were measured by systems serology and reported as $\log_{10}$ mean fluorescence intensity (MFI). Pregnant women (n=7) are shown in orange and non-pregnant females (n=13) in purple. Triangles highlight women who received monovalent boosters and circles represent bivalent booster recipients. Horizontal bars indicate mean MFI across samples with bars indicating standard error of the mean (SEM). **(A)** IgG subtype abundance to ancestral nucleocapsid (N) protein**.** IgG subtypes, including **(B)** IgG1 **(C)** IgG2, **(D)** IgG3, and **(E)** IgG4, in response to ancestral, BA.5, XBB.1.5, and JN.1 spike protein. Data were analyzed by linear mixed effects regression analysis with Bonferroni post-hoc. Asterisks indicate $p < 0.05$.

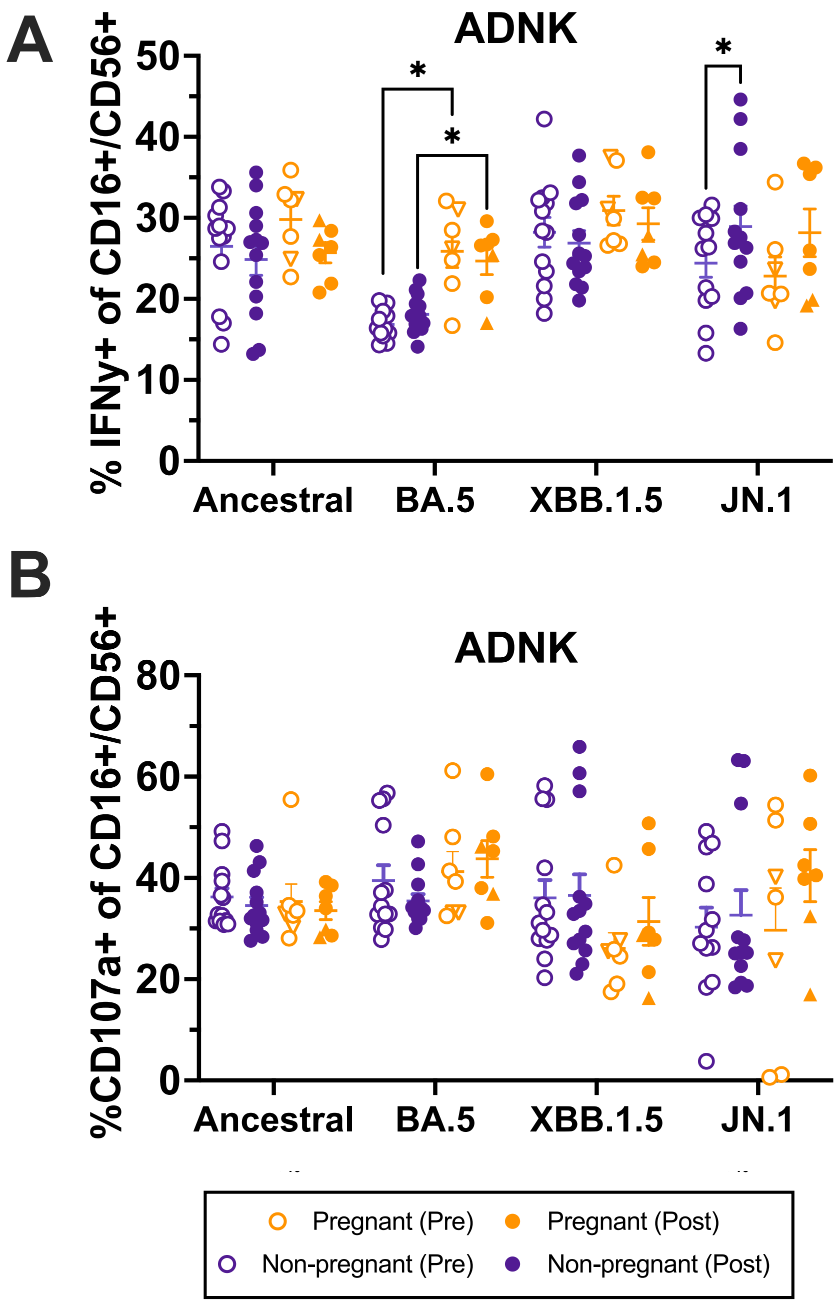

Supplemental Figure 2. Additional antibody-dependent NK cell responses by pregnancy status. NK cells isolated from donor blood were utilized to assess antibody dependent NK cell cytotoxicity (ADNK), or the ability of antigen specific serum antibody to include NK cell cytokine production or degranulation in serum pre and 3-5 weeks post-vaccination. Pregnant women (n=7) are shown in orange and non-pregnant females (n=13) in purple. Triangles highlight women who received monovalent boosters and circles represent bivalent booster recipients. Horizontal bars indicate mean MFI across samples with bars indicating standard error of the mean (SEM). ADNK was measured as ability to induce **(A)** IFNy production or **(B)** NK cell degranulation, as measured by CD107a positivity. Data were analyzed by linear mixed effects regression analysis with Bonferroni post-hoc. Asterisks indicate $p < 0.05$.

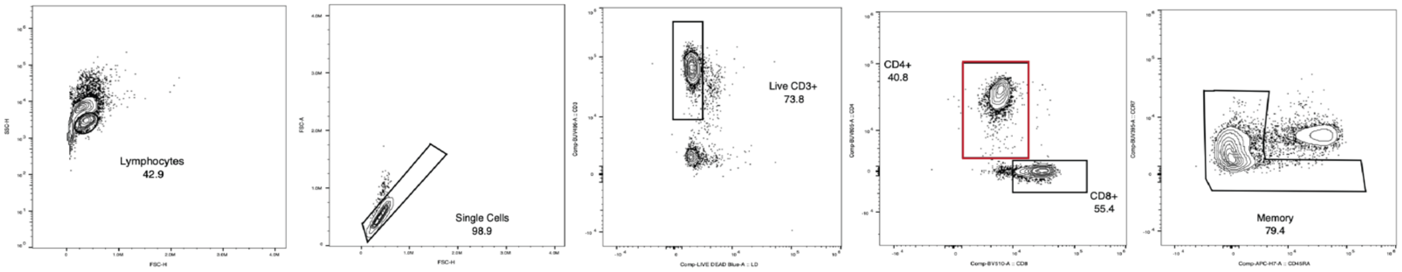

Supplemental Figure 3A. Gating strategy for CD4⁺ memory T cells. Representative flow cytometry gating of spike-specific memory CD4+ T cells

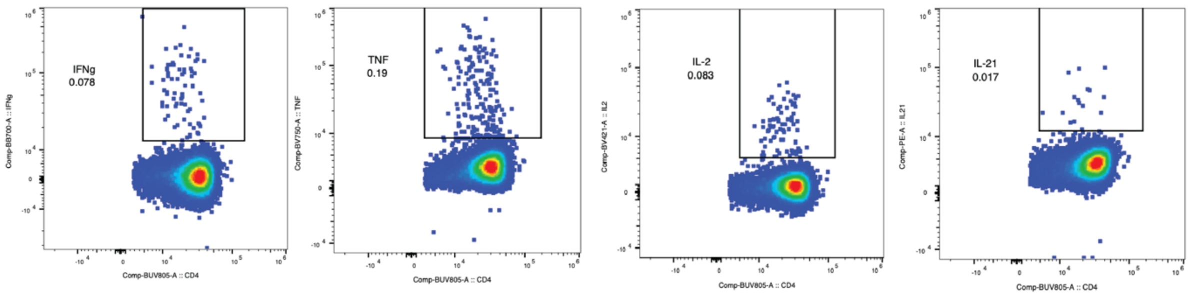

Supplemental Figure 3B. Cytokine positive CD4⁺ T cells. Representative flow cytometry gating of cytokine (IFNg, TNF, IL-2, and IL-21) producing spike-specific memory CD4+ T cells.

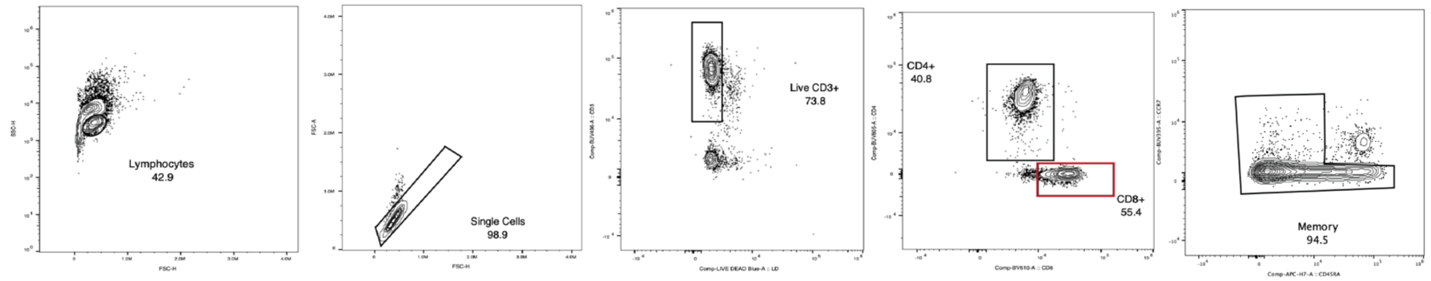

Supplemental Figure 4A. Gating strategy for CD8⁺ memory T cells. Representative flow cytometry gating of spike-specific memory CD8+ T cells

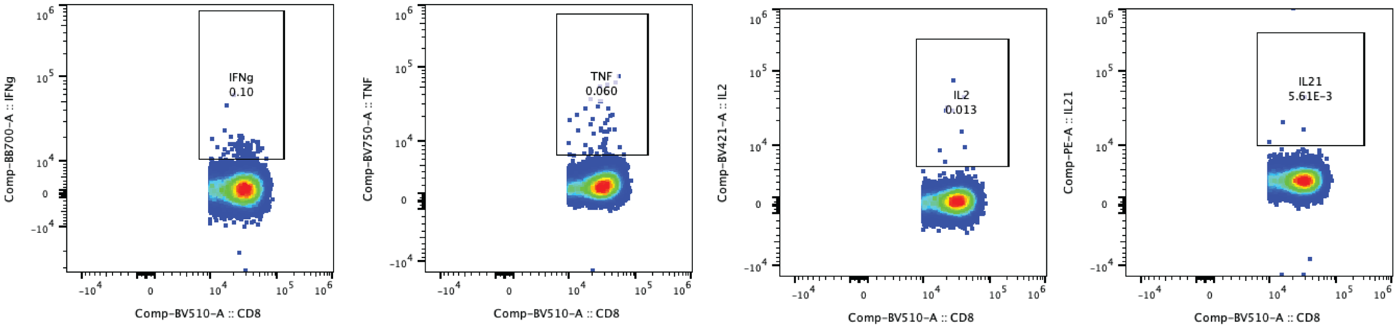

Supplemental Figure 4B. Cytokine positive CD8⁺ T cells. Representative flow cytometry gating of cytokine (IFNg, TNFa, IL-2, and IL-21) producing spike-specific memory CD8+ T cells.

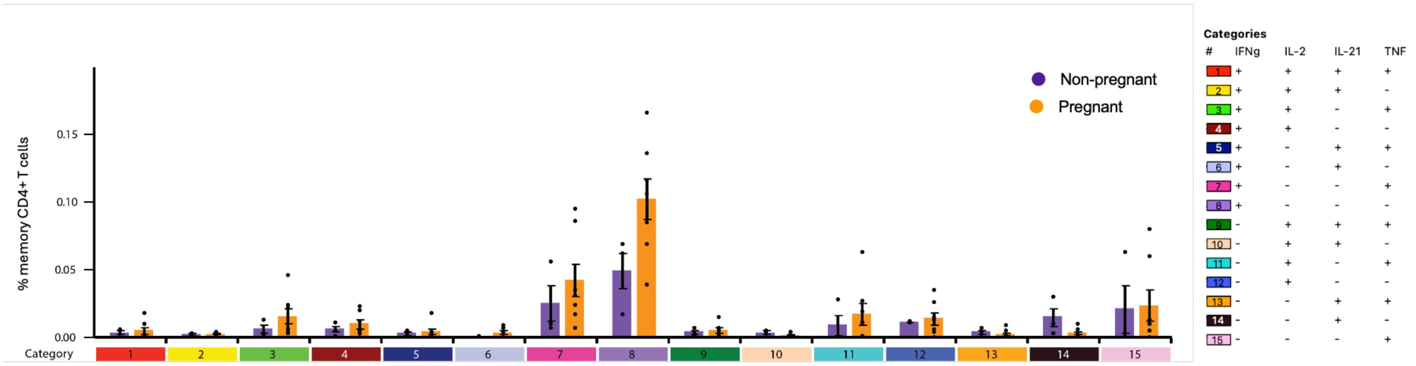

Supplemental Figure 5. Pre- vs post-vaccination CD8+ T cells in pregnant and non-pregnant women. Peripheral blood mononuclear cells (PBMCs) were collected pre and 3-5 weeks post-vaccination for flow cytometry analyses. Cytokine-producing, antigen-specific CD4+T cells for **(A)** pregnant (n=7) and **(B)** non-pregnant (n=3) women by the 15 cytokine combination categories. Histograms show the percent of memory CD4+ T cell producing cytokines following stimulation with ancestral SARS-CoV-2 spike peptide pools post-vaccination. Bars reflect mean values with standard error of mean.

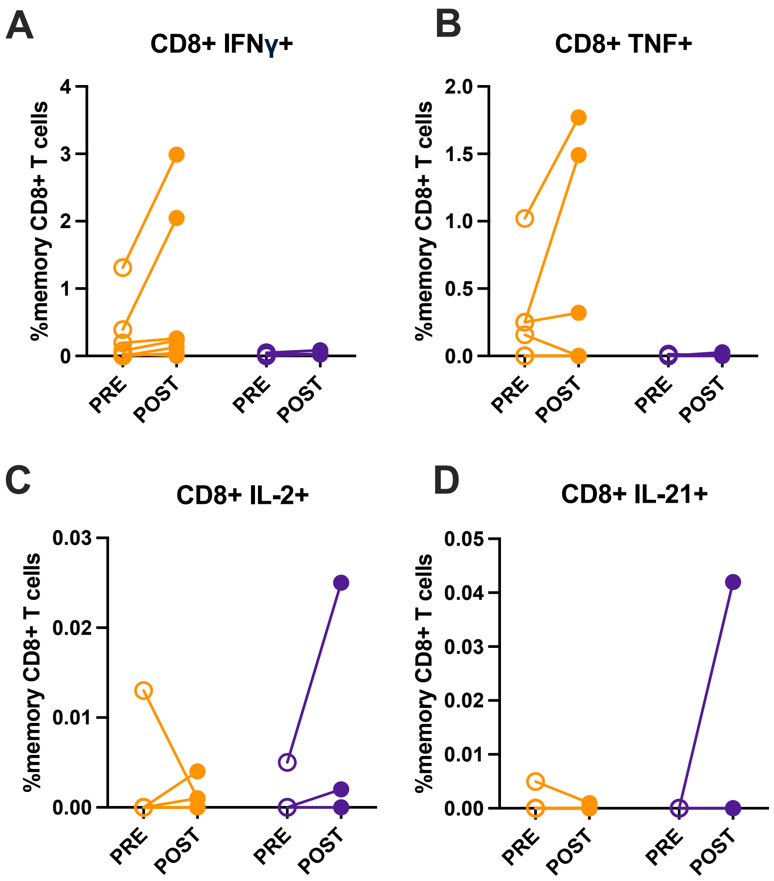

Supplemental Figure 6. Pre- vs post-vaccination CD8+ T cells in pregnant and non-pregnant women. Peripheral blood mononuclear cells (PBMCs) were collected pre and 3-5 weeks post-vaccination for flow cytometry analyses. Paired dot plots demonstrate the percent of memory CD8+ T cell producing (A) IFNγ, (B) TNF, (C) IL-2, or (D) IL-21 following stimulation with ancestral SARS-CoV-2 spike peptide pools pre- and post-vaccination.

Supplemental Table 1. Intracellular cytokine staining (ICS) flow cytometry panel. (A) surface staining markers and **(B)** intracellular markers.

A. Surface

Fluorophore	Marker	Clone	Catalog	Vendor
BUV395	CCR7	2-L1-A	749655	BD Biosciences
BV650	CXCR3	1C6	740603	BD Biosciences
PE CF594	KLRG1	2F1	565393	BD Biosciences
BV605	CD69	FN50	562989	BD Biosciences
BUV563	CD25	2A3	612918	BD Biosciences
BUV661	PD-1	EH12.1	750260	BD Biosciences
BUV737	TIM-3	7D3	748820	BD Biosciences
BV570	CD28	CD28.2	302926	Biolegend
PE Cy5	CD127	HIL-7R-M21	BD Custom	BD Biosciences
BUV805	CD4	SK3	612887	BD Biosciences
APC H7	CD45RA	HI100	560674	BD Biosciences
BB660	Tigit	741182	BD Custom	BD Biosciences
BV711	OX40	ACT35	563664	BD Biosciences
BV786	CD27	L128	563327	BD Biosciences
PE Cy7	CTLA-4	BNI3	BD Custom	BD Biosciences
BV510	CD8a	HIT8a	300934	Biolegend
BB790	CXCR5	RF8B2	BD Custom	BD Biosciences

B. Intracellular

Fluorophore	Marker	Clone	Catalog	Vendor
PE	IL-21	3A3-N2	513004	BioLegend
AF680**	HK2	EPR20839	ab228819	Abcam
AF488	TCF1	812145	IC8224G	FisherScientific
BV421	IL-2	MQ1-17H12	564164	BD Biosciences
AF532***	VDAC1	20B12AF2	ab14734	Abcam
BV750	TNF	MAb11	566359	BD Biosciences
PECy5.5*	CPT1a	8F6AE9	ab128568	Abcam
AF405	Tomm20	EPR15581-54	ab210047	Abcam
Alexa647	GLUT1	EPR3915	ab195020	Abcam
BUV496	CD3	UCHT1	612940	BD Biosciences
BB700	IFNy	B27	566394	BD Biosciences

Notes: * conjugated using PE/Cy5.5 Conjugation Kit - Lightning-Link (ab102899)

** conjugated using DyLight 680 Conjugation Kit (Fast) - Lightning-Link (ab201804)

*** conjugated using Alexa Fluor 532 Antibody Labeling Kit (Thermo Fisher, A20182)
